# Mitochondria power the nucleus under pressure

**DOI:** 10.1016/j.mbm.2025.100146

**Published:** 2025-08-12

**Authors:** Meng Yao, Yao Zong, Junjie Gao

**Affiliations:** a Institute of Microsurgery on Extremities, And Department of Orthopedic Surgery, Shanghai Sixth People's Hospital Affiliated to Shanghai Jiao Tong University School of Medicine, Shanghai, 200233, China; b Centre for Orthopaedic Research, Medical School, The University of Western Australia, Nedlands, WA, 6009, Australia

**Keywords:** Mechanical confinement, Mitochondrial dynamics, Nuclear ATP

## Abstract

Mechanical confinement of cells, as occurs during processes like tumor cell invasion or immune cell trafficking, poses a pressure that can threaten nuclear integrity and cell viability. Recent findings illuminate a rapid adaptive mechanism by which cells under acute compressive stress rearrange their internal architecture to preserve nuclear functions. Upon confinement, mitochondria swiftly relocate to cluster around the nucleus (forming nuclear-associated mitochondria, NAM), entrapped by a web of endoplasmic reticulum (ER) and actin filaments. This proximity provides a localized surge of ATP within the nucleus, fueling energy-intensive nuclear processes, notably maintaining an open chromatin state and facilitating efficient DNA damage repair. This targeted energy delivery maintains nuclear chromatin accessibility, supports DNA repair mechanisms, and ensures sustained cell proliferation despite physical constraints. Here we provide a commentary on these findings, discussing the biological significance of mitochondria–nucleus repositioning, the role of nuclear ATP in safeguarding chromatin, and the broader implications for cellular fitness in development and disease.

Perturbed mitochondrial dynamics can compromise nuclear ATP availability under mechanical stress, thereby endangering genomic integrity. Ghose et al. investigated how mitochondria mitigate this issue: by modulating their dynamics, such as perinuclear clustering or enhanced cristae organization, they sustain a robust nuclear ATP supply even under confinement.^1^ This crosstalk underscores mitochondria as not merely energy producers but active regulators of nuclear resilience, with their dynamics serving as a bridge between mechanical cues and nuclear energy homeostasis. Elucidating these mechanisms provides critical insights into how cells withstand mechanical challenges, with implications for developmental biology, disease pathogenesis, and tissue engineering.

Cells are perpetually subjected to mechanical forces exerted by the extracellular matrix (ECM), and maintaining nuclear homeostasis under such mechanical confinement demands precise energy regulation.^2^ Central to this process is the dynamic crosstalk between mitochondria and the nucleus, where mitochondrial function directly influences nuclear processes dependent on ATP.^3^ Mechanical confinement, including spatial restriction or increased ECM stiffness, elicits adaptive alterations in mitochondrial dynamics.^4^ Nuclear ATP, the primary energy currency for chromatin remodeling, transcription, and DNA repair, is predominantly generated via mitochondrial oxidative phosphorylation.^5^ Above all, the link between mitochondria-derived nuclear ATP and mechanical confinement is truly remarkable. Through a PubMed search, we found that nuclear ATP, mitochondrial dynamics, and mechanical confinement had a strong inclusiveness from 2000 to 2025 (Fig. 1). Therein, the most notable point was not until 2025 that the relationship among these three entities was clarified innovatively.

**Fig. 1:**
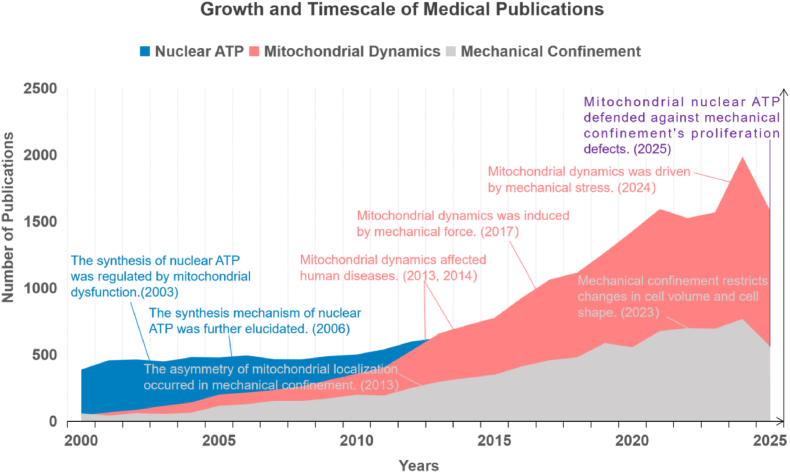
Growth and timescale of medical publications on nuclear ATP, mitochondrial dynamics, and mechanical confinement. This figure illustrated the number of published papers over time, related to nuclear ATP, mitochondrial dynamics, and mechanical confinement, along with significant milestones from 2000 to 2025 (by Aug 6, 2025). The data were visualized through three-layered area graphs, each representing a different category of publications: Research on nuclear ATP (blue area), mitochondrial dynamics (pink area), and mechanical confinement (gray area), and purple represented the combination of the three entities. This graph underscored the expanding research and clinical importance of mitochondria-derived nuclear ATP linked with mechanical confinement. The x-axis represented the years, ranging from 2000 to 2025, and the y-axis quantified the number of published papers.

## 1 Mechanotransduction and metabolic adaptation

Cells in vivo often encounter spatial confinement and compressive forces during normal physiology and pathology. For example, cancer cells navigating through a crowded tumor microenvironment or entering/exiting blood vessels face extreme physical constraints, as do immune cells squeezing through tissues and cells during inflammation. Even in development, rapid cell migrations such as those in gastrulation expose cells to acute mechanical stress. These physical challenges can deform the cell and nucleus, disrupt cytoskeletal organization, and alter chromatin architecture – potentially leading to DNA damage and mitotic errors. Remarkably, however, cells often continue to survive and divide under such stresses. This suggests the existence of adaptive mechanisms that preserve nuclear homeostasis – i.e., the stability of nuclear structure and function – in the face of mechanical deformation.

Previous studies of mechanotransduction have illuminated the nucleus sensed mechanical challenges and acted as an intracellular ruler to measure cellular shape variations.^6^^,^^7^ The nuclear envelope provides a gauge of cell deformation and activates a mechanotransduction pathway that controls actomyosin contractility and migration plasticity. By contrast, Ghose et al. focused on the immediate cellular response to acute mechanical confinement.^1^ They posed a critical question: beyond well-known long-term responses, do cells rapidly rewire organelle organization and metabolism to maintain nuclear functions under sudden physical stress? To address this, the authors developed an in vitro model of acute cell confinement, compressing cells to a height of ∼3 μm using an agarose overlay, which mimics transient spatial constraints encountered during invasion or migration. This brief compression (15 minutes) was sufficient to cause cytoskeletal reorganization and membrane blebbing, confirming the induction of mechanical stress.

Using subcellular fractionation and proteomic analysis, mechanical confinement causes a relocation of mitochondria to the nuclear vicinity. Mitochondrial proteins became strongly enriched in the nuclear fraction of confined cells compared to unconfined controls. High-resolution microscopy confirmed that, whereas unconfined cells show virtually no mitochondria near the nucleus, confined cells rapidly accumulate mitochondria around the nuclear periphery. This accumulation was observed not only in cultured cells but also in patient-derived tumor samples, indicating that it represents a physiologically relevant phenomenon in vivo. Ghose et al. termed these perinuclear mitochondrial clusters NAM (nuclear-associated mitochondria) and noted that their formation produces distinctive nuclear indentations as the clustered mitochondria press into the nucleus.^1^ Crucially, the mitochondria remain outside the nuclear membrane (not entering the nucleus), nestling into grooves at the nuclear surface. This relocalization is critical for providing direct ATP supply to the nucleus during acute mechanical stress, highlighting a previously underappreciated metabolic adaptation strategy in response to cellular confinement.^8^^,^^9^

## 2 Mitochondrial relocation during mechanical confinement

Under confinement, the reorganization of mitochondria around the nucleus is both rapid and highly orchestrated. Mitochondrial repositioning is an active process requiring the cytoskeletal and ER networks.^10^^,^^11^ Ghose et al. demonstrate an extensive ER meshwork around the nucleus acts as a scaffold or “net” that traps mitochondria at the nuclear periphery, and this entrapment is actin-dependent.^1^ Pharmacological disruption of F-actin polymerization (using Latrunculin A) greatly reduced NAM formation, indicating that de novo actin polymerization is required to drive mitochondria into the perinuclear region. Notably, inhibiting actin filament elongation via formin inhibition (SMIFH2) mimicked the effect of Latrunculin, whereas blocking Arp2/3-mediated actin branching did not, pinpointing the importance of linear actin cables in this process. Stabilizing actin filaments with Jasplakinolide also prevented NAM formation, further emphasizing that dynamic, remodeling actin – rather than pre-existing filaments or passive trapping – underlies the mitochondrial movement. In contrast, depolymerizing microtubules with Nocodazole did not inhibit NAM; in fact, it slightly enhanced mitochondrial clustering under confinement. This suggests that microtubules may normally distribute mitochondria throughout the cytoplasm, and their removal shifts the balance toward actin-driven perinuclear clustering. Indeed, microtubule breakdown likely impairs mitochondrial fission, favoring more fused mitochondria that are captured by the actin–ER network. Together, these findings support a model in which the actin cytoskeleton and ER jointly mediate NAM formation, while the microtubule network and mitochondrial fission/fusion state modulate how extensively mitochondria accumulate around the nucleus.

Mitochondrial morphology was found to be an important factor in this mechanistic interplay. Cells genetically engineered for elongated, fused mitochondria (e.g., FIS1 knockout, which inhibits fission) showed significantly higher NAM levels under confinement than cells with fragmented mitochondrial networks (e.g., MFN1 knockout, which inhibits fusion). Fused mitochondria, being more interconnected, may be more effectively retained by the perinuclear ER/actin scaffold, and they also carry a higher capacity for ATP production. Consistently, fused networks led to a “bolder” nuclear ATP surge upon confinement, whereas fragmented mitochondria produced a weaker response. These observations align with the idea that cells under stress benefit from maintaining a contiguous mitochondrial network to maximize local energy delivery.

Structurally, the NAM clustering causes transient nuclear deformation. Mitochondria accumulate above and below the nucleus, creating indentations that give the nucleus a characteristic multi-lobed or “doughnut/bean”-shaped appearance. Importantly, preventing NAM formation (e.g., with actin inhibitors) also prevented these nuclear indentations, indicating that the physical presence of mitochondria is what molds the nuclear shape. The nucleus is the cell's largest organelle and usually resists deformation, but here it is pliable enough to accommodate incoming mitochondria. While this nuclear indentation might initially seem detrimental, Ghose et al. found it to be coupled to a crucial metabolic advantage: mitochondria positioned in these nuclear pockets can directly fuel the nucleus with ATP.^1^ The NAM architecture, therefore, appears to be an adaptive structural change – essentially repositioning the “powerhouses” of the cell in direct proximity to the nucleus when mechanical stress needs it.

## 3 Nuclear ATP surge and chromatin accessibility

One of the most remarkable consequences of NAM formation is a rapid surge of ATP within the nucleus during confinement, which is directly attributable to mitochondrial activity: when cells were treated with oligomycin (to block ATP synthase) or with a mitochondrial uncoupler (BAM15), the confinement-induced increase in nuclear ATP was effectively abolished. Likewise, disrupting actin polymerization or ER–mitochondria contacts prevented the ATP surge, consistent with those perturbations blocking NAM formation. These interventions establish that nucleus-proximal mitochondria are the source of the ATP, and that their physical clustering (mediated by actin/ER) is required for the energy transfer to the nucleus. Notably, the study found that providing cells with excess metabolic substrate (pyruvate) could partially rescue the nuclear ATP levels even when mitochondrial ATP synthase was inhibited. This implies that cells possess a metabolic flexibility to buffer nuclear ATP – for instance, by upregulating glycolysis or importing extracellular nutrients – if oxidative phosphorylation is compromised, highlighting the priority placed on sustaining nuclear energy under stress.

Why is a nuclear ATP influx so beneficial? The nucleus houses many ATP-dependent processes, including chromatin motion, transcription, and DNA repair.^12^^,^^13^ Ghose et al. provide evidence that the confinement-triggered ATP surge helps maintain chromatin in an open, accessible state, which is crucial for these processes.^1^ In their experiments, acutely confined cells with an intact ATP surge showed relatively stable chromatin accessibility, whereas confined cells treated with oligomycin (no ATP surge) exhibited a pronounced chromatin compaction. Genome-wide assays of chromatin accessibility (via ATAC-seq) revealed that blocking mitochondrial ATP production during confinement led to reduced accessibility across the genome, particularly in gene-rich chromosomes. Many of the regions that became less accessible were associated with genes involved in actin cytoskeleton remodeling and cell cycle regulation. By contrast, control confined cells (with a normal ATP surge) maintained higher accessibility at those same genomic regions. Thus, the mechano-induced nuclear ATP appears to facilitate the loosening of chromatin or prevent its compaction, enabling continued expression of genes needed for cytoskeletal dynamics and cell cycle progression even under compression.

This link between ATP and chromatin is consistent with the known requirement of energy-consuming remodelers to reposition nucleosomes and expose DNA. Indeed, ATP-dependent chromatin remodeling complexes are essential for efficient DNA damage repair because they open up chromatin to allow repair factors access to broken DNA. Fittingly, Ghose et al. discovered that the nuclear ATP surge has a direct impact on the DNA damage response during confinement. Acute compression of cells was found to induce some DNA double-strand breaks (evidenced by 53BP1 and γH2AX foci), likely due to mechanical distortion of the nucleus. However, if the nuclear ATP surge was blocked, the cells failed to properly form 53BP1 repair foci in response to the damage. In other words, without ATP, the DNA breaks were not recognized and organized into repair centers, presumably because the chromatin remained too condensed or immobile to recruit repair machinery. This phenomenon was not unique to mechanical stress – the authors showed that even DNA damage caused by chemicals (etoposide) or radiation produced minimal 53BP1 focus formation when ATP synthesis was simultaneously inhibited. Metabolomic data further supported that DNA repair is an energy-intensive process: cells recovering from DNA damage showed depletion of ATP pools unless new ATP was continuously generated. Together, these findings highlight that the surge of nuclear ATP is functionally necessary to promote a robust DNA repair response under confinement stress. By fueling chromatin remodeling at damage sites, the ATP ensures that repair proteins can access DNA lesions and resolve them efficiently.

In summary, mechanical confinement triggers a mechano-metabolic signal – the clustering of mitochondria and consequent ATP influx to the nucleus – that guards the epigenetic and genetic stability of the cell. The confined nucleus uses this ATP to keep chromatin primed for rapid gene activation and for swift DNA repair signaling. Intriguingly, they observed a feedback loop: many of the genes that lost accessibility when ATP was absent are involved in actin dynamics. This suggests that without the ATP surge, cells may fail to properly deploy actin-based structural changes needed for stress adaptation, creating a vicious cycle. Conversely, when ATP is plentiful, chromatin remains accessible at these loci, enabling the cell to transcribe and reorganize the actin cytoskeleton for reinforcement. In essence, the actin cytoskeleton helps generate a nuclear ATP boost, and that ATP in turn helps maintain actin-related gene programs, forming a positive feedback loop supporting cellular resilience.

## 4 Implications for proliferation and cellular fitness

The ultimate payoff of this confinement-induced mitochondrial repositioning is an improvement in cell survival and proliferative capacity under otherwise growth-restrictive conditions. By safeguarding DNA integrity and the transcription of cell-cycle genes, the nuclear ATP surge helps cells to continue cycling after compression. Ghose et al. demonstrated this by tracking cell cycle progression in cells released from confinement. Cells that had experienced acute confinement (15 min) but had an intact nuclear ATP response were able to resume proliferation with minimal delay – their S-phase cells completed DNA replication and progressed to the next cell cycle phase on a timescale comparable to unconfined cells. In contrast, cells whose ATP surge was pharmacologically blocked showed a pronounced delay in S-phase progression after confinement. Many of these cells remained stuck in S-phase or took much longer to divide, indicating that unresolved DNA damage or inaccessible chromatin had impeded their replication cycle. By 36 hours after confinement, <3 ​% of control (ATP-competent) cells were still in S-phase, whereas a significantly larger fraction of oligomycin-treated (ATP-depleted) cells was unable to exit S-phase. This translated into reduced proliferation: overall cell numbers and cell cycle completion rates were lower when the nuclear ATP surge was suppressed. These data clearly support that the confinement-induced ATP surge is crucial for maintaining cell fitness, allowing cells to overcome the mechanical stress without permanent cell cycle arrest. In practical terms, the mitochondria-derived ATP burst gives cells a better chance to repair any DNA damage and properly duplicate their genome, thereby preventing mechanical stress from causing genomic instability or cell death.

An important question is whether this mechanism operates in physiological contexts beyond the artificial compression assay. The authors provide evidence that it does: observations in human tumors revealed cells with perinuclear mitochondrial clustering in situ. In stained sections of breast carcinomas, about 1.8 ​% of tumor cells in the densely packed core regions exhibited the NAM phenotype, while at the invasive front (where cells infiltrate surrounding tissue) the incidence was higher, around 5.4 ​%. Although a small subset, these NAM-positive cells in vivo likely represent those experiencing acute mechanical confinement (for example, cells squeezing through stromal barriers or pores in the matrix). The enrichment of NAM at the tumor invasive edge suggests that this adaptation might confer a survival or growth advantage to cells confronting physical barriers during invasion. By clustering mitochondria and fueling the nucleus, invasive tumor cells could better tolerate the DNA damage and replication stress associated with migration through tight spaces, thereby maintaining their proliferative capacity even as they disseminate.

The ability to proliferate under confinement has clear implications for cancer progression, as tumors often grow in confined, high-pressure environments.^14^^,^^15^ It may also be critical for processes like metastasis (circulating tumor cells enduring shear forces and capillary constrictions) and for immune cells that must remain functional after traversing basement membranes. More broadly, any cell that must be divided within a mechanically stressed environment would benefit from this mechanism. Without it, mechanical forces can trigger cell cycle arrest or apoptosis – phenomena that have been documented in spheroid models and other systems when compressive stress is unmitigated. Thus, the confinement-induced nuclear ATP surge can be viewed as a buffering system against mechanical stress, ensuring that short-term physical insults do not translate into long-term proliferative defects.

In conclusion, Ghose et al. unveils a rapid mechano-metabolic adaptation that allows cells to withstand mechanical confinement by reallocating resources at the subcellular level.^1^ The mechanical stress of confinement, rather than halting cell growth, triggers mitochondria to rally around the nucleus and supercharge it with ATP. This ingenious cellular tactic ensures that even under physical compression, the nucleus remains operational – DNA is faithfully repaired, and critical genes remain active – so that the cell can continue its mission to divide and thrive. It is still worth investigating how universal is the NAM response among different cell types and organisms? Could chronic confinement (as in fibrosis or growing tumors) induce longer-term metabolic or epigenetic reprogramming via this mechanism? And importantly, what sensors and signals coordinate the dance between the cytoskeleton, mitochondria, and nucleus under force?

## CRediT authorship contribution statement

**Meng Yao:** Writing – original draft. **Yao Zong:** Writing – review & editing. **Junjie Gao:** Supervision, Project administration, Funding acquisition, Conceptualization.

## Ethical approval

This study does not contain any studies with identifiable human or animal subjects performed by any of the authors.

## Declarations of competing interest

No competing interest of all authors.
